# Comparative Analyses of Plant Transcription Factor Databases

**DOI:** 10.2174/138920209787581253

**Published:** 2009-03

**Authors:** Silvia R Ramirez, Chhandak Basu

**Affiliations:** School of Biological Sciences, University of Northern Colorado, Greeley, Colorado, USA

**Keywords:** Plant transcription factor databases, bioinformatics.

## Abstract

Transcription factors (TFs) are proteinaceous complex, which bind to the promoter regions in the DNA and affect transcription initiation. Plant TFs control gene expressions and genes control many physiological processes, which in turn trigger cascades of biochemical reactions in plant cells. The databases available for plant TFs are somewhat abundant but all convey different information and in different formats. Some of the publicly available plant TF databases may be narrow, while others are broad in scopes. For example, some of the best TF databases are ones that are very specific with just one plant species, but there are also other databases that contain a total of up to 20 different plant species. In this review plant TF databases ranging from a single species to many will be assessed and described. The comparative analyses of all the databases and their advantages and disadvantages are also discussed.

## INTRODUCTION

Transcription factors (TFs) play many important roles in plant developmental processes and plant responses to the change in environments [1]. The complete sequencing of *Arabidopsis* *thaliana* [2] and the rice (*Oryza sativa*) [3] genomes have propelled the ongoing research on plant molecular genetics and genomics. There are many new and powerful genomic tools [4] to power the research in the field of plant genomics and proteomics. It is crucial that important information is gathered about the plant cellular, biochemical, and molecular processes that drive the responses to the change in the environment in plants, and TFs databases are precisely a tool that aids in this ongoing research. Databases for *Arabidopsis thaliana *are most extensive than for any other plant. In this review, we presented a comparative analysis of several publicly available plant TF databases. To use the information presented here most effectively, the readers are encouraged to browse the respective TF database websites when reading this article. All website names, database and bioinformatics tools abbreviations mentioned throughout this paper have been explained in Table **1** unless they are explained in parentheses. This review will explore different TF databases and assess overall strengths and weaknesses of each and Table **2** summarizes the overviews of the plant TF databases.

## RARTF: RIKEN *ARABIDOPSIS* TRANSCRIPTION FACTOR DATABASE (JAPAN)

The RARTF: RIKEN *Arabidopsis* Transcription Factor Database (http://rarge.gsc.riken.jp/rartf/) is a database only for *Arabidopsis* *thaliana *TFs [5]. This database is organized in table like manner including the TF ID, TF family, and the number of TF members in each family. The website has a position specific interative BLAST or PSI-BLAST search feature which allows to search for distantly related protein families. If specifically looking for a TF of *Arabidopsis thaliana* then this database should be a great resource. However, if searching for a database that can compare across different species, then it would not be as helpful. The table setup at the opening page of the website is user friendly because all TF family can be browsed and accessed from the main homepage. In addition to that, the other search tools are right below the TF table where they are easily seen. The content of the website includes the table of all TFs, reverse PSI-BLAST search, BLAST search, PSI- BLAST search, and retrieve FASTA format sequence file. Some of the great features of the database are inclusion of the motif of TFs and full-length cDNAs associated with each gene coding for the TF. If a TF is selected from the database table, it redirects to another table that contains the gene IDs that then provide gene code, gene name, e-value, transposon mutants, RAFL, and genome map. In addition to the well organized data provided, the website contains direct links to other plant genome information websites/databases like TIGR, TAIR, and MIPS. It should be mentioned here that The Institute of Genomic Research (TIGR), USA has merged with J. Craig Venter Research Institute (JCVRI), USA. So, it is time to the change the TIGR link to JCVRI link. The minor error that needs attention is when the reverse PSI-BLAST link is selected an error message appears which states, “You don't have permission to access /rartf/rps_blast/ on this server”. The website does not have downloads which are part of other databases, and there is no link to directly contact the developers if needed. On the main homepage there are additional links to the RIKEN *Arabidopsis *Genome Encyclopedia: RARGE and to the *Arabidopsis *Gene Regulatory Information Server: agris (Ohio State University, USA). The website was last updated in July 20, 2006. Overall this is an effective database for *Arabidopsis thaliana *TFs.

## PLANT TRANSCRIPTION FACTOR DATABASES (CHINA)

The Plant Transcription Factor Databases (http://planttfdb.cbi.pku.edu.cn/) [6] provide information for putative TFs of the following plant species, namely, *Arabidopsis thaliana, Populus trichocarpa, *and *Oryza sativa*. It also contains TF sequence information of *Chlamydomonas reinhardtii, *and* Physcomitrella patens*. Additionally it contains databases based on the available EST sequences from 17 other plant species: (maize, barley, wheat, sorghum, sugarcane, upland cotton, soybean, potato, apple, sweet orange, grape, sunflower, barrel medicago, lotus, loblolly pine, and white spruce). In the main homepage there is introductory information that familiarizes the viewer with what is offered by the database. Strengths of this database are it includes important information about each TF family and goes well in depth as far as giving the functional domains for each TF. Another positive aspect of this database is it includes citations associated with each individual TF family. This database takes it a step further by displaying TF entries of what other databases like UniProt, RefSeq, and TransFac have. Information such as TF family and associated genes can be accessed by selecting a plant species and then can be browsed by family or by chromosome. Once the TF family is selected the next choice is by gene. The gene information consists of some basic information, gene structure, annotation, protein sequence features, 3D structure, ontology, expression, sequence, and references on other databases. All the information given by selecting each gene is very well organized and visually appealing to the viewer. In the main homepage, it is important to note that each plant species has a database and a page of its own that opens when selected. The BLAST option will provide different types of blast searches like blastp, blastn, blastx, and tblastn. For each BLAST option, it is possible to choose one of the 22 plant species. The search option takes to sub-databases by selecting specific species. A great asset of this website is the fact that it has a large number of different species that can be viewed and selected even if 17 of them do not have a complete genome sequenced. Available downloads include protein sequences files, CDS (coding sequence) sequences files, and ortholog prediction files. The overall website is well organized, and maintained; last revised in May 14, 2008.

## PLANT TRANSCRIPTION FACTOR DATABASE (GERMANY) 

The Plant Transcription Factor Database (http://plntfdb.bio.uni-potsdam.de/v2.0/) [7] provides TF data for three plant species (*Oryza sativa subsp. japonica,* *Arabidopsis thaliana, *and *Populus trichocarpa*) and for *Chlamydomonas reinhardtii*, *Cyanidioschyzon merolae*, *Ostreococcus tauri, *and *Physcomitrella patens* also. The database compiles a total of 68 TF families. The outline of the homepage is a table of all the TF families followed by a table of other transcriptional regulators. It is also possible to search the database by sequence identifier or by gene ID. When selecting one of the seven plant species link, it will go directly to a page in which it contains information on TF families associated with that particular plant species. For that given family of TFs there are series of links that reveal important information such as gene model, TF description and domain. Additionally the database also provides information about genome databases, orthologs and co-orthologs, domain architecture (start codon, stop codon, and e-value), protein, and transcription sequences associated with each TF family. Another way the database can be used is through the table of TFs family that lead to a description of the family characteristics and a list of all the species that contain this TF family. The website allows for BLAST search through the PubMed engine. The download link could be used to search for protein sequence, TF domain architecture, transcription sequence, and a list of TFs. The most convenient use of this database is being able to select a specific TF family and find which plant species is included in that TF family. It has a great verity of plant species that include rhodophyte, prasinophyte, chlorophyte, bryophyte, monocot, and eudicot. A minor pitfall in the database is the website has no indication of when it was last updated which tend to be a useful fact to know in plant genomics field which is moving at accelerated speed when it comes to new data publications. Overall this site is very effective in providing a good sample of plant TFs from several plant species that are not common amongst other databases.

## PLANT CARE (BELGIUM) 

The Plant Cis-Acting Regulatory Element (CARE) or Plant CARE (http://bioinformatics.psb.ugent.be/webtools/plantcare/html/) [8] is a database that contains data accessible freely for academia users. The site contains 435 plant transcription sites from monocots, dicots and other plants. Additionally more than 159 plant promoters are described. The main homepage include a ‘Query Care search’ for information on a *cis*-acting regulatory site. The user can search broadly or narrowly for information by changing search parameters when performing the search. Another option is searching for *cis-*acting regulatory sites present in promoter sequences. Other query options include searching for TFs from supergroups (e.g. eukaryotes) to all the way down to a species (e.g. *Arabidopsis*). One can search by gene name or by name of a TF. A search can be done under ‘Referencia’ for TF related papers relevant to Plant CARE. Some of the strengths of this database are the new features it offers, a ‘Motif sampler’, which consists of searching for regulatory element in a dataset of co-regulated promoter sequences, and also includes clustering of microarray [9] data. When comparing this database with others, it can be rated as a useful one if looking for information for monocot, dicot, and more specifically *cis*-acting regulators. The database however does not list names of all the TFs like other databases. One of the weaknesses of this database is it does not list specifically what gene families or plant species form part of the TF database; first the user needs to search for a TF and then only the user knows what plant species is included in that particular TF family. The website does not have a feature to download or provide links to other databases. The link ‘Enter new data’ is also a non-functional link.

## PLACE (JAPAN) 

The PLACE or Plant *cis*-acting Regulatory DNA Elements database (http://www.dna.affrc.go.jp/PLACE/index. html) [10] consists of plant *cis*-acting regulatory DNA elements mainly for vascular plant only, but the database has recently started to include *cis* elements information from *Chlamydomonas reinhardtii* too. Overall the website is very clearly put together which ensures quick navigation. The following searches are possible: by keyword, homology search by FASTA formats etc. The signal scan search and homology search can be used to find motifs identical or similar to previously reported *cis* element motifs. Nucleotide sequences can be entered (or copied and pasted) in this database by selecting the signal scan search file upload tab and the resulting page will predict and show all putative TFs for that sequence. The database is a good resource to find *cis* elements. One of the most attractive features is the way the database is maintained up to date by inclusion of relevant research papers and adding them to the database. The database does not have download features but gives information on where they can be obtained. A big disadvantage of this database is the database maintenance was permanently ceased on February 2007. This database would be helpful if the researcher is looking for plant *cis *acting elements and already have a gene ID, PubMed ID numbers, or GenBank nucleotide sequence ID numbers. As time progresses the database will become eventually outdated, but at this moment it still contains very relevant and useful information.

## DATABASE OF *ARABIDOPSIS* TRANSCRIPTION FACTORS (DATF) (CHINA) 

The Database of Arabidopsis Transcription Factors (DATF) (http://datf.cbi.pku.edu.cn/) [11] is entirely dedicated to *Arabidopsis thaliana* TFs. Some of the best features of the database include the classification of all TFs into 64 families which are accessible from the homepage of the website. The family organization of all the TFs has multiple elements of the DNA-binding domain and neighbor-joining phylogenetic trees of each family. Similarly to other databases this one contains a homolog tab with the rice TF database DRTF (discussed later). Additionally it contains information of about 1200 TFs, protein domains, and 3D structure information. One can search by either TF family or by chromosome. There is a link from the homepage on how to access other databases such as rice (DRTF), poplar (DPTF), and Plant Transcription Factor Databases, China ( http://planttfdb.cbi.pku.edu.cn/). Information provided for a given TF family consists of family introduction, family structure, and family binding sites. The following information can be downloaded: sequences of the family genomic sequence, CDS sequence, and protein sequence. Under the specific TF family, it is also possible to generate phylogenetic trees with genes in each TF family using the ‘Phylogenetic Tree JPG Image’ tool. One can also align the amino acid sequences encoded by genes of a TF family by choosing the ‘Multi-alignment’ option. A list of the total family ‘loci’ and ‘gene model’ are listed under each family. The gene models are organized in a table setup similar to the family class, making it an easy way to look through the information. This is a great database but its weaknesses are that it is only for one plant species and the last update for the database was in July 2006. Overall it would be considered an effective database used for *Arabidopsis thaliana*.

## THE DATABASE OF POPLAR TRANSCRIPTION FACTORS (DPTF) (CHINA) 

The Database of Poplar Transcription Factors (DPTF) [12] (http://dptf.cbi.pku.edu.cn/) is dedicated to TFs of the black cottonwood tree poplar (*Populus trichocarpa*). The homepage gives basic information on the database such as the total (2576) number of TFs gene models, and the number of TF families (64). Strengths of this database are it is configured in a way that similar searches can be made against major databases (e.g. UniProt, RefSeq, EMBL, TRANSFAC) and EST information gathered from numerous microarray [9] experiments are also available to provide the most accurate information about all the putative TFs. Like other databases this database also includes multiple alignment of DNA-binding domain of each TF family, neighbor-joining phylogenetic tree of each family, and the homolog searches with the Database of *Arabidopsis* Transcription Factors (DATF). The database can be explored by using the TF family table or by the chromosome table. The putative TF gene models (or putative mRNAs synthesized by the genes) in *Populus trichocarpa *are included in each table*.* Under the gene family tab, there is a list of genes organized in a table like manner. The link LinkOut will directly take the viewer to one of the following databases: JGI, DATF, and DRTF. Additionally, like DATF database, other tools like TF gene alignments, phylogenetic tree drawings etc. are available from each TF family page. The page was last updated in May 2008. The database is frequently being maintained and updated. The speed and diligence with which this is done is vital to provide information that will advance the knowledge in the field. The search page gives the viewer the capability to search by database ID (or gene ID) or description, by homology relationship (e.g. compare with DATF), and by expression information (e.g. in which plant tissues). The BLAST search allows the user to search a TF amino acid sequences and compare the TF sequences with other plant TF databases like *Arabidopsis* and rice.

## THE DATABASE OF TOBACCO TRANSCRIPTION FACTORS (TOBFAC) (USA) 

The database of tobacco transcription factors (http://compsysbio.achs.virginia.edu/tobfac/) [13] contains a total of 65 TF families organized in a table like format. The available genes are noted next to the TF family name. The database contains a total of over 2513 tobacco TFs. The feature ‘Blast against Tobacco’ consists of selecting what kind of program the viewer is interested in using (blastp, blastn, blastx, tblastn, and tblastx) and what database (*Nicotiana tabacum*, EST, Genbank, UniGenes, Genbank, and TOBFAC transcription factor sequences) would someone like to use. One can use gene sequence ID, DNA or protein sequence to search for TFs. The ‘EST tobacco Blast’ results generated a total of 572 EST sequences (TOBFAC ID, accession, e-value, definition or information of the EST etc.). The Gen-Bank protein BLAST results in 2354 BLAST outputs, which also have TOBFAC ID, accession, e-value etc. A list of published papers on each TF family is available under the list of papers options. This feature is especially helpful if someone is doing research on a particular TF family. The see all search queries tab provides a list of the TF families with a description of their 3D structures and the protein sequences. The published genes tab gives a list of the TF family, Gen-Bank ID, name, and the PMID (PubMed citation ID) for all published genes. The GenBank ID takes the viewer directly to the NCBI webpage for the nucleotide sequence. The database also allows comparing TOBFAC sequences with Gen-Bank ID side by side by generating a spreadsheet. Some of the great features of this database are that sequences can be queried by BLAST searches, and also downloaded for further analysis. Similarly to other databases TOBFAC has tools to generate phylogenetic trees for TF families. The database is frequently updated and well maintained with it last update dated on January 2008.

## ATHAMAP (GERMANY) 

The AthaMap (http://www.athamap.de/) [14] is a database dedicated solely to *Arabidopsis thaliana* and contains a total of 109 TFs, potential TFs, and small RNA binding sites in *Arabidopsis thaliana *genome. A list of the 109 TFs can be located under the documentation tab. There are three main useful tools on the AthaMap website: search, colocalization, and gene analysis; all of these can be accessed by using the tool tab. The power of the search tool consists of being able to submit genomic position and get a TF binding site. This can be accomplished by entering genomic location or the *Arabidopsis* Genome Initiative (AGI) number. The colocalization tab provides the ability to identify colocalization of TF binding sites of pairs of TFs included in the AthaMap database. The gene analysis tool generates graphical representation of binding of many TF families with respect to any *Arabidopsis* gene. The overall setup of the website is very organized and helpful. Some of the weaknesses of the database are as follows. It is not possible to compare the database to other TF databases. Downloads are not possible which would have been very helpful. The TF coverage (109) is rather low compared to other databases. The strengths of the database are that it has the ability to provide information on potential TFs and small RNA binding sites, which are not common in other databases. It was last updated in June 2008, which is a great indication that it is a database that is often updated and well maintained. Overall this is a well put together website but it is very specific as far as what it offers.

## DBD: TRANSCRIPTION FACTOR PREDICTION DATABASE (UK) 

The DBD: Transcription factor prediction database (http://www.transcriptionfactor.org) [15] contains the most extensive selection when it comes to the total number of genomes (927) and the capacity to search for predicted TFs sites. The database has the capability to search by genomes list, or by TF families list. The Browse Genomes tab provides names of the genomes, kingdoms, genomic sizes, number of available TFs etc. Selecting any given organism’s genome will redirect to a page with list of genes and their corresponding TF domains for the genes. The Browse Family tab includes a table with TF Family name, Family ID, HMM (Hidden Markov Model) ID [16], predicted TF domain architecture, and taxonomic distribution. The taxonomic distribution link is an interesting and useful feature in this database. The taxonomic distribution link generates a graphical map of taxonomic distribution of a particular TF across the kingdom. This database is one of the most ambitious in providing an extensive and well-put together database for TFs from 927 genomes. The strengths also include the fact that the website was just updated in July 2008 which is a clear indication of how well maintained and up-to-date the information is. The impressive features of this database are different types of search parameters that can be used to conduct a search (e.g. sequence ID, gene name, SUPERFAMILY, Pfam, or by organism and DNA-binding domain name). The overall database and website are one of the best plant TF databases.

## THE DATABASE OF RICE TRANSCRIPTION FACTORS (DRTF) (CHINA) 

The Database of Rice Transcription Factors (DRTF) (http://drtf.cbi.pku.edu.cn/) [17] is a TFs database for *Oryza sativa* L. ssp. *Indica* and *Oryza sativa* L. ssp. *Japonica*. This database contains information on 2025 putative (TF) gene models in *Oryza sativa* L. ssp. *Indica* and 2384 putative (TF) gene models in *Oryza sativa* L. ssp. *Japonica* and covers 63 TF families. Each TF family has a detail summary of the important information about the TF family along with a list of the genes, and domain alignment. The peptide sequence and CDS sequence downloads are available for both *indica* and *japonica*. The database shares homology with both the *Arabidopsis* (DATF) and *Populus* (DPTF) transcription factor databases. A list of the family ‘loci’ and ‘gene model’ are available under each family. Gene models have a table setup similar to the family class, allowing for easy navigation. ‘Gene model’ offers: basic information, and a link to DPTF (discussed earlier). General structure of the website consist of a direct link to mother database Plant Transcription Factor Database (http://planttfdb.cbi.pku.edu.cn), gene structure, annotation, protein sequence features, 3D structure, ortholog, expression, and sequence. This is a great database but its weaknesses are that it is only for one plant species and the last update for the database was in 2006. Overall it would be considered an effective database used for rice research.

## CONCLUSION

TFs play an important role gene expression in plants and other organisms. TFs have an active role in initiating the transcription process as well as regulating the development and response to environmental signals throughout the life of the organism. TFs trigger cascades of biochemical reactions altering the gene expression profile of a plant cell. In plants, especially, it is important to understand how TFs binding along with gene expression synchronize downstream chemical reactions. These reactions help plants respond to the challenging environmental conditions since plants are sessile and cannot escape from environmental stresses. TF databases are surely a valuable tool for plant biologists to study plant physiology, plant developmental biology and plant stress physiology. The researchers have options of choosing a particular plant TF database for a single plant species. Alternatively one can choose a TF database, which can compare several TFs from various plant species side by side. All the databases will be enriched in future with completion of several other plant genome sequences. Plant TF databases are a vital part in studying the roles of TFs as well as aid in discovering new cell functions. The wide array TF databases freely available over the Internet are definitely a valuable resource for plant molecular biologists. It is important that the respective database developers update the databases frequently. Some databases may have some limitations, but there is no doubt that these TF databases will open new avenues of research in the fields of plan molecular biology, plant genomics and plant biotechnology.

## Figures and Tables

**Table 1:** Information on Several Databases, Institutes, and Bioinformatics Tools

Name of the Database, Bioinformatics Tool or Institute	Relevant Website Address	Comment and Relevant References
AGRIS (Arabidopsis Gene Regulatory Information Server)	http://arabidopsis.med.ohio-state.edu/	Database for *Arabidopsis *promoter sequences and TFs and their target genes [18]
BLAST (Basic Local Alignment Search Tools)	http://blast.ncbi.nlm.nih.gov/Blast.cgi	Comparison of nucleotide or amino acid sequences from various organisms [19]
blastn	http://blast.ncbi.nlm.nih.gov/Blast.cgi	Comparison of nucleotide sequence against nucleotide sequence query
blastp	http://blast.ncbi.nlm.nih.gov/Blast.cgi	Comparison of a protein query against a protein database
blastx	http://blast.ncbi.nlm.nih.gov/Blast.cgi	Comparison of a protein database using a translated nucleotide query
CBI	http://www.cbi.pku.edu.cn/	First bioinformatics center in China
EMBL (The European Molecular Biology Laboratory)	http://www.embl.de	EMBL is an international intergovernmental (Germany, UK, France, and Italy) research organization in Europe promoting molecular biology and genomics research [20]
EST (Expressed Sequence Tags)	www.ncbi.nlm.nih.gov/dbEST/	A EST database is made from randomly sequenced cDNA clones [21, 22]
E-value	http://blast.ncbi.nlm.nih.gov/Blast.cgi	E-value determines the stringency of a blast search, lower e-value means good match with the query sequence and an e-value of 0 (zero) means 100% match with the query sequence
FASTA	http://www.ncbi.nlm.nih.gov/blast/fasta.shtml	It is a text format used to write protein or nucleotide sequences
GenBank	http://www.ncbi.nlm.nih.gov/Genbank/	One of the largest genetic sequence databases containing records of 85,759,586,764 bases (as of September 4, 2008) [23]
IPGC (International Poplar Genome Consortium)	http://www.ornl.gov/sci/ipgc/	A consortium for post genomic sequence research on poplar [24]
JGI (Joint Genome Institute)	http://www.jgi.doe.gov/	A US Dept. of Energy institute promoting research on genomics of various organisms
MIPS (Munich Information Center for Protein Sequences)	http://mips.gsf.de	A database for nucleotide and protein sequences [25]
NCBI	http://www.ncbi.nlm.nih.gov/	National Center for Biotechnology Information, Bethesda, Maryland, USA
Pfam	http://pfam.sanger.ac.uk/	A database of proteins families represented by multiple sequence alignments and hidden Markov models [26]
Position-Specific Iterative BLAST (PSI-BLAST)	http://www.ncbi.nlm.nih.gov/Education/BLASTinfo/psi1.html	Search for distantly related protein families
PubMed	http://www.ncbi.nlm.nih.gov/pubmed/	A database of over 18 million citations
RAFL (RIKEN Arabidopsis full-length (RAFL) cDNA)	http://rarge.gsc.riken.jp/cdna/cdna.pl	A database to search for *Arabidopsis* full length cDNAs [27]
RARGE (RIKEN Arabidopsis Genome Encyclopedia)	http://rarge.gsc.riken.jp/	A database for *Arabidopsis* genome [28]
RefSeq (The Reference Sequence collection)	http://www.ncbi.nlm.nih.gov/RefSeq/	A database of well annotated DNA, protein and transcript sequences developed from genomic information from diverse organisms [29]
RIKEN	http://rarge.gsc.riken.jp/index.html	A research foundation in Japan
SUPERFAMILY	http://supfam.org/SUPERFAMILY/	A database containing structural and functional protein annotations from over 900 organisms [30]
TAIR (The *Arabidopsis* Information Resource)	http://www.arabidopsis.org	A comprehensive resource for *Arabidopsis* genome [31]
tblastn	http://blast.ncbi.nlm.nih.gov/Blast.cgi	Comparison of a protein query sequence against a nucleotide sequence database
tblastx	http://blast.ncbi.nlm.nih.gov/Blast.cgi	tblastx converts a nucleotide query sequence into protein sequences and the resulting sequences are compared to search for homologous regions
TIGR (The Institute for Genomic Research)	http://www.tigr.org/	Previously known as The Institute for Genomic Research, currently known as the J. Craig Venter Institute located in Rockville, Maryland, USA
TRANSFAC	http://www.gene-regulation.com/pub/databases.html	TF database [32]
UniGene	http://www.ncbi.nlm.nih.gov/UniGene/	A database of transcriptome of many organisms, maintained by NCBI [33]
UniProt (The Universal Protein Resource)	http://www.uniprot.org/	Protein sequence database [34]

**Table 2:** Comparison of Several Plant Transcription Factor Databases

Name of the Database and Website Address	Country of Origin	Plant Species Covered	Can the Database be Accessed Browsing Individual Chromosome?	Can the User Search by Gene ID or Sequence Identifier?	Is Data Download Possible?	Was the Database Updated in 2007?
RARTF (http://rarge.gsc.riken.jp/rartf/)	Japan	*Arabidopsis thaliana*	No	No	No	No
Plant Transcription Factor Databases (http://planttfdb.cbi.pku.edu.cn/)	China	*Arabidopsis thaliana, Populus trichocarpa, Oryza sativa*	Yes	No	Yes	Yes
Plant Transcription Factor Database (http://plntfdb.bio.uni-potsdam.de/v2.0/)	Germany	*Oryza sativa subsp. japonica, Arabidopsis thaliana, *and *Populus trichocarpa*	No	Yes	Yes	No information available
Plant CARE (http://bioinformatics.psb.ugent.be/webtools/plantcare/html/	Belgium	Many plant species	No	Yes	No	No
PLACE (http://www.dna.affrc.go.jp/PLACE/index.html)	Japan	Vascular plants and *Chlamydomonas reinhardtii*	No	Yes	No	Yes
DATF (http://datf.cbi.pku.edu.cn/)	China	*Arabidopsis thaliana*	Yes	Yes	Yes	No
DPTF (http://dptf.cbi.pku.edu.cn/)	China	*Populus trichocarpa*	Yes	Yes	No	Yes
TOBFAC (http://compsysbio.achs.virginia.edu/tobfac/)	USA	*Nicotiana tabacum*	No	Yes	Yes	Yes
Athamap (http://www.athamap.de/)	Germany	*Arabidopsis thaliana*	Yes	Yes	No	Yes
DBD: Transcription factor prediction database (http://www.transcriptionfactor.org)	UK	*Arabidopsis thaliana, Zea mays, Oryza sativa*, *Populus trichocarpa*, *Sorghum bicolor*, *Vitis vinifera*, *Lotus japonicus*	Yes	Yes	Yes	Yes
DRTF http://drtf.cbi.pku.edu.cn/	China	*Oryza sativa*	Yes	Yes	Yes	No
